# Intraoperative catheter directed thrombolytic therapy for the treatment of superior mesenteric and portal Vein thrombosis

**DOI:** 10.1016/j.ijscr.2018.09.030

**Published:** 2018-10-05

**Authors:** M. Marshad, M. Maresch, T. Al Abbasi

**Affiliations:** a Bahrain Defence Force Hospital, West Riffa, Bahrain; b BDF Hospital, Bahrain

## Abstract

**Introduction and case presentation:**

Acute portal and superior mesenteric vein thrombosis(SMV) is a rare but potentially lethal condition that is often characterized by generalized and non-specific symptoms. A high index of suspicion is warranted for early diagnosis and management. We present a case of 54 year old male who presented with generalized abdominal pain which was later accompanied by hemodynamic instability and radiological diagnosis of portal vein and superior mesenteric vein thrombosis.

**Discussion:**

The management of SMV can be divided into medical and surgical therapy. Patients who have clear signs of peritonitis will require emergent surgery. Interventions for life-threatening portal vein thrombosis are limited and poorly described in the literature.

**Conclusion:**

We report a case of extensive portal vein thrombosis(PVT) advancing proximally to involve the superior mesenteric vein. Rapid portal vein patency and improved hepatic function was achieved with the direct use of tissue plasminogen activator infusion via operatively placed middle colic vein catheter.

## Highlights

• Acute portal and superior mesenteric vein thrombosis (SMV) are a rare but potentially lethal conditions.
• Superior Mesenteric Vein Thrombosis is accompanied by non-specific symptoms, making the diagnosis rather challenging.
• The initial management of SMV thrombosis usually involves the initiation of anticoagulants.
• Operative management is reserved for underlying bowel compromise where peritoneal signs are exhibited.

## 1 Introduction

Thrombosis involving the confluence of SMV (superior mesenteric vein) and PV (portal vein) is an uncommon condition associated with hypercoagulable states, intra-abdominal infections, inflammation and trauma. PV and SMV thrombosis may occur as a consequence of medical conditions such as cirrhosis, pancreatitis or surgical interventions such as a splenectomy [1]. PVT may extend proximally to involve the SMV and splenic vein. The condition is often accompanied by non-specific symptoms, making the diagnosis rather challenging. Patients may be asymptomatic, though many manifest numerous non-specific symptoms including abdominal pain, nausea, anorexia and unintentional weight loss. When the thrombus extends to the SMV it may be associated with a 20% risk of mesenteric ischemia and <10% risk of infarct [2,3]. Where symptoms of peritonitis exist, there should be a high index of suspicion for bowel infarction and perforation. The mortality rate associated with mesenteric vein thrombosis(MVT) remains approximately 20–50% [4]. The treatment of mesenteric and portal vein thrombosis may be divided into medical and surgical management. In the absence of an acute surgical abdomen, non-operative management is the mainstay.

We present a case of PVT and mesenteric vein thrombosis and attempted management with direct intraoperative middle colic vein thrombolytic infusion.

*The following case report has been reported in line with SCARE criteria* [5].

## 2 Case

A 54 year old male, with no comorbidities or significant social history presented to the emergency department with a three day history of intermittent generalized abdominal pain radiating to the back. He did not endorse a history of constitutional symptoms. Upon presentation to the emergency department he was hemodynamically stable. Physical examination revealed a soft abdomen with generalized tenderness particularly in the epigastric region. Initial lab results showed a WBC 6.77 × 109/L, serum amylase 79IU/L, urinary amylase 738IU/L, CRP 73.88 mg/L, D-dimer 13.66ug/ml, and a Lactate of 1.87 mmol/L. The patient was subsequently admitted under the gastroenterology service with the diagnosis of pancreatitis.

**Fig. 1:**
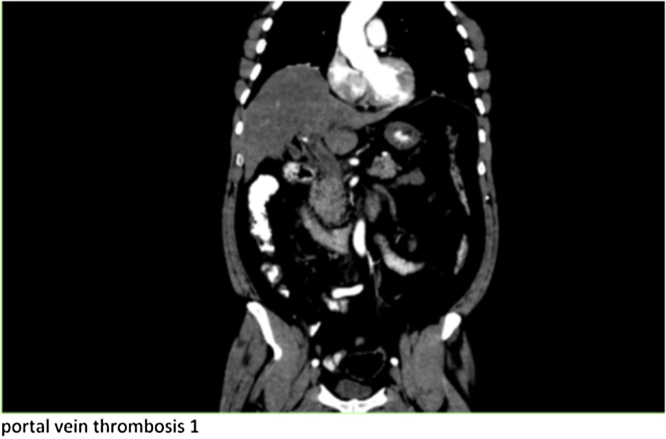
xxx.

**Fig. 2:**
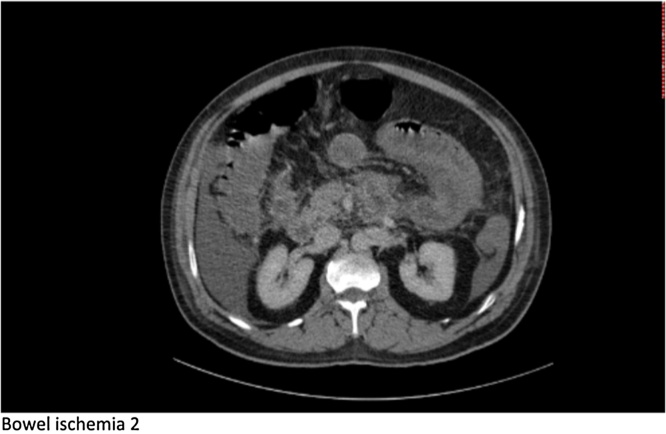
xxx.

**Fig. 3:**
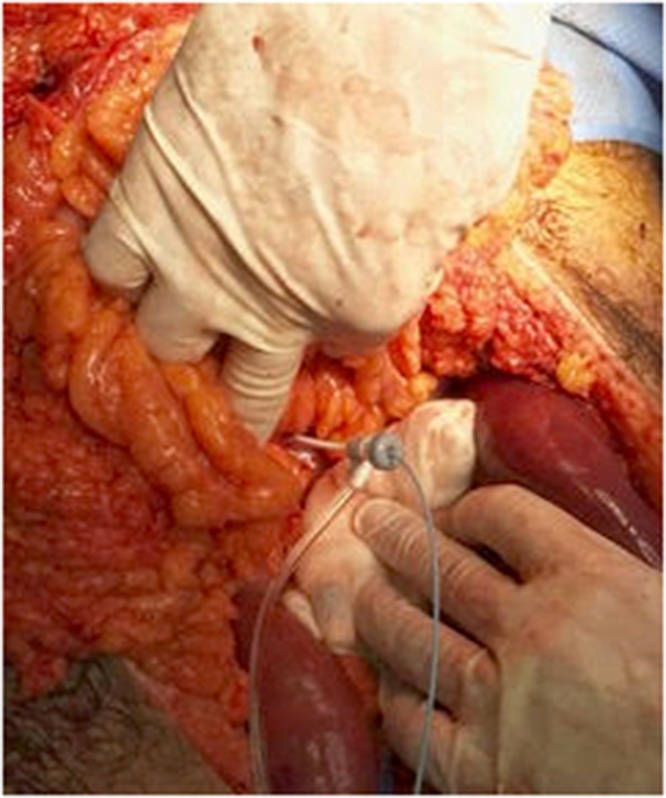
xxx.

Preliminary CT Abdomen with oral and IV contrast showed acute pancreatitis with superior mesenteric and portal vein thrombosis and no evidence of mesenteric small bowel involvement (Fig. 1). He was managed conservatively on the ward by means of heparin Infusion and thereafter switched to LMWH and warfarin. A week later he developed sustained tachycardia and sudden drop in WBC with associated increase in lactate to 5.31IU/L. A follow up CT abdomen with IV contrast revealed diffuse wall thickening involving multiple loops of jejunum highly suspicious of bowel ischemia (Fig. 2). A decision was made to proceed with an exploratory laparotomy which revealed no evidence of full thickness necrosis but characteristics of small bowel edema secondary to SMV thrombosis. Additionally, the pancreas appeared inflamed and edematous with evidence of intraperitoneal calcification within the lesser sac. An ABthera vacuum dressing was applied in anticipation of a second look laparotomy. The patient was shifted to the ICU where he showed evidence of progressive acute hepatic failure with rising INR, bilirubin, and serum ammonia level. On re-evaluation of the bowel within 48 h, we noted viable but dusky appearing jejunum. An access sheath was inserted retrograde from the middle colic vein into the superior mesenteric vein and portal vein where a catheter was placed for the direct thrombolysis (Fig. 3). A bolus of Heparin 1000 IU followed by 500 IU/hr infusion was delivered through the sheath along with Altepase 1 mg/hr for a 24 h period.

**Fig. 4:**
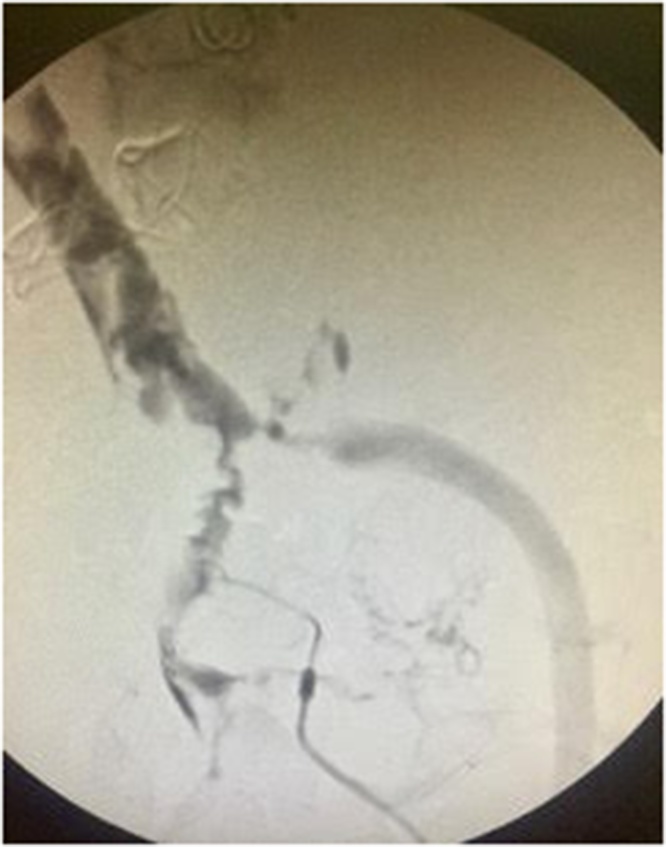
xxx.

Following the 24 h period, a third look exploratory laparotomy was performed where the vascular catheter placed was removed. An Intraoperative venography showed patent portal veins (Fig. 4). However, 100 cm segment of proximal bowel was found ischemic and therefore a resection and side to side anastomosis was done.

The patient had an uneventful post-operative course. A week after his final laparotomy, an ultrasound abdomen was performed showing resolved portal and superior mesenteric vein thrombosis. The patient was discharged in a satisfactory condition on Warfarin 3 mg PO OD.

## 3 Discussion

Simultaneous acute thrombosis of the portal and superior mesenteric vein is an uncommon but potentially lethal condition. A type IV PVT is a condition whereby thrombosis extends proximally to involve the superior mesenteric and splenic vein. This type of classification is associated with a worse prognosis [6]. A high index of suspicion coupled with prompt diagnosis and management is crucial for survival. D-dimer is a sensitive but non-specific serum marker for the condition [7]. Non-operative management of MVT may entail non-invasive or invasive techniques. Non-invasive techniques involve the utilization of the traditional systemic anticoagulation to prevent further thrombosis and thrombus propagation or combined therapy of anticoagulant and thrombolysis; for clot dissolution. Although the systemic anticoagulation regimen has been shown to be effective, its rates of recanalization are less likely in comparison to intravascular thrombolysis [8]. One complication that emerges due to the failure of recanalization with the use of anticoagulants includes the development and rupture of varices [9]. The following complication is a consequence of increased pressure in retrograde vessels at areas of portosystemic shunting (esophageal, gastric, duodenal and splenic veins) which dilate and form torturous venous channels. More invasive managements involve catheter-directed thrombolysis. Due to the isolated character of the porto-mesenteric bed it is rather complex to direct the catheter into the thrombus. Transjugular-transhepatic approach has been reported as successful in some cases. Furthermore, percutaneous transarterial approach into SMA with hope for transcapilary distribution of thrombolytic agent has shown sporadic success [10,11].

The initial management of SMV thrombosis usually involves initiation of anticoagulants. Operative management is reserved for underlying bowel compromise where peritoneal signs are exhibited. The mainstay intraoperative management is dependent on the surgical findings. Where infarction exists the management entails bowel resection. SMV thrombosis with underlying infarction is associated with a 50% mortality rate [12]. In our case we were forced by the circumstances to go for a laparotomy to assess bowel viability. This gave us the chance to directly access mesenteric veins and place the catheter into the thrombus itself for effective thrombolysis.

We believe that our patient developed portal vein thrombosis that extended proximally to the superior mesenteric vein resulting in mesenteric ischemia and subsequent infarction as well as acute hepatic failure. The use of direct intravenous catheterization and thrombolytic infusion has the advantage of direct clot lysis. This was evident by biochemical and radiological parameters. Upon instillation of thrombolysis, liver function tests improved and a repeated doppler ultrasound performed two weeks post procedure revealed a patent portal and superior mesenteric vein with adequate venous flow.

During the outpatient department follow up on the patient, it was found that his IgG4 levels were elevated, 1360 [Normal Range 0.039–0.0864] which satisfies one of the criteria suggestive of autoimmune pancreatitis [13].

Although systemic anticoagulation with heparin is the traditional therapy for acute portal and superior mesenteric vein thrombosis, catheter directed thrombolysis may be a resourceful adjunct in the management of this potentially lethal condition. Early Surgical Intervention with resection of infarcted bowel followed by post-operative anticoagulant therapy is crucial for promising outcomes.

## Conflicts of interest

The following is a disclosure that the following case report holds no financial or otherwise personal relationship with other people or organizations that may influence our work. No conflict of interest is present.

## Sources of funding

The following manuscript did not involve any funding resource for any part of the process.

## Ethical approval

Research approval has been attained by the hospital body ethics committee, furthermore consent for writing up the case report while maintaining anonymity was taken from the patient.

## Consent

Written informed consent was obtained from the patient for publication of this case report and accompanying images. A copy of the written consent is avaialble for review by the Editor-In-Chief of this journal on request.

## Author contribution

Writing the paper: Dr. Maha Marshad.

Data Collection: Dr. Maha Marshad.

Data Analysis: Dr. Maha Marshad, Dr. Thamer Al Abbasi.

Literature Research: Dr. Maha Marshad.

Manuscript Editing and Review: Dr. Thamer Al Abbasi, Dr. Martin Maresch.

Manuscript Finalization: Dr. Maha Marshad, Dr. Thamer Al Abbasi.

## Registration of research studies

N/A.

## Guarantor

Dr. Maha Marshad.

## Provenance and peer review

Commissioned, externally peer-reviewed.
